# A test of desert shrub facilitation via radiotelemetric monitoring of a diurnal lizard

**DOI:** 10.1002/ece3.4673

**Published:** 2018-11-16

**Authors:** Michael F. Westphal, Taylor Noble, Harry Scott Butterfield, Christopher J. Lortie

**Affiliations:** ^1^ US Bureau of Land Management Central Coast Field Office Marina California; ^2^ Department of Biology York University Toronto Ontario Canada; ^3^ The Nature Conservancy San Francisco California

**Keywords:** animal interactions, ectotherm, endangered species, *Ephedra californica*, *Gambelia sila*, plant, San Joaquin Desert, thermoregulatory behavior

## Abstract

Preservation of desert ecosystems is a worldwide conservation priority. Shrubs can play a key role in the structure of desert communities and can function as foundation species. Understanding desert shrub ecology is therefore an important task in desert conservation. A useful model for the function of shrubs in deserts is ecological facilitation, which explores benefits that shrubs confer on their community. Facilitation has been well developed in the context of shrub–plant interactions but less well studied for plant–animal interactions. We used radiotelemetry to test the hypothesis that a dominant desert shrub facilitates one species of diurnal lizard. We hypothesized that the blunt‐nosed leopard lizard *Gambelia sila* would spend some part of its daily activity cycle associated with California jointfir *Ephedra californica*, and that lizard association with shrubs would increase during the afternoon peak temperature period. We relocated lizards three times daily for 24 days and scored whether lizards were within 0.5 m of a shrub, which we used as an indicator of shrub association. For each relocation, we also scored lizard association with a set of predefined microhabitat features. We also scored lizard behavior according to a set of predefined behavioral traits. We constructed home ranges following the minimum convex polygon method and generated estimates of shrub density and relative shrub area within each home range polygon. We obtained 1,190 datapoints from a sample of 27 lizards. We found that lizards were associated with open sites significantly more often than with shrubs but were associated with shrubs more than predicted by percent shrub area within their home ranges. Lizards were associated significantly more often under shrubs during the afternoon peak temperature period, and lizards were observed cooling under shrubs significantly more often. The frequency of association of individual lizards with shrubs was not correlated with the density of shrubs within their home range. *Synthesis and Applications*. Shrubs can be considered as a component of high‐quality habitat for ectothermic desert vertebrates for the purposes of restoration and management. Furthermore, radiotelemetry provides a novel methodological approach for assessing shrub–animal facilitative interactions within desert communities.

## 1 INTRODUCTION

Deserts are highly distinct ecosystems that contribute significantly to global biodiversity and global ecosystem function. The conversion and loss of desert habitat is therefore a global biodiversity crisis requiring immediate intervention, including conservation of remaining undisturbed habitat and restoration of degraded desert (Bachelet, Ferschweiler, Sheehan, & Strittholt, 2016; Cook, 2004; Hannah, Carr, & Lankerani, 1995; Hoekstra, Boucher, Ricketts, & Roberts, 2005; Kéfi et al., 2007; Mouat & Lancaster, 2008; Westphal, Stewart, Tennant, Butterfield, & Sinervo, 2016). Identifying the drivers of ecological health in desert communities will be a crucial component of such interventions. Shrubs can maintain the diversity of desert plant communities (Flores & Jurado, 2003) and are predicted to play significant roles in the thermal ecology of desert ectotherms (Basson, Levy, Angilletta, & Clusella‐Trullas, 2017; Sears et al., 2016). Shrubs can also facilitate ectotherm populations in the face of climate change (Adolph, 1990; Kearney, Shine, & Porter, 2009; Sears & Angilletta, 2015; Sears et al., 2016; Sinervo et al., 2010).

**Figure 1:**
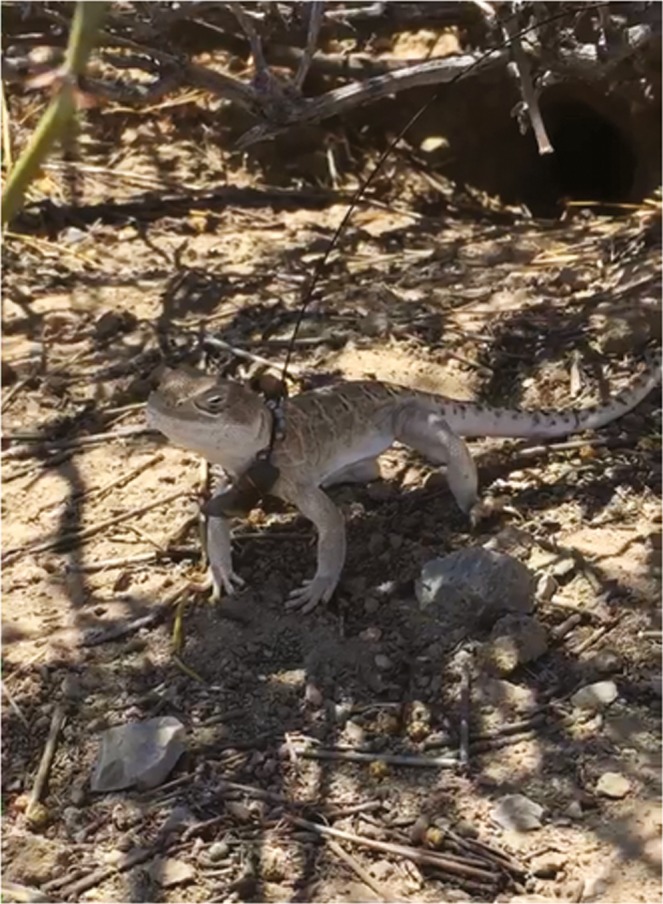
A radio‐collared blunt‐nosed leopard lizard, *Gambelia sila*, stands under the canopy of a California jointfir, *Ephedra californica*

Ecological facilitation theory provides a roadmap for describing and predicting the beneficial interactions of shrubs with other organisms within their communities (Bruno, Stachowicz, & Bertness, 2003; Bulleri, Bruno, Silliman, & Stachowicz, 2016; Filazzola & Lortie, 2014; Filazzola, Westphal, et al., 2017; Mcintire & Fajardo, 2014). Using facilitation theory, Filazzola, Westphal, et al. (2017) extended the exploration of the beneficial interactions between desert shrubs and vertebrates and found that one species of shrub provided facilitative benefits to a target species of lizard. We sought to confirm and add depth to their findings using radiotelemetry tracking of the same target species (Figure 1). Radio telemetry is a well‐tested and powerful tool that allows the longitudinal tracking of individual animals throughout their daily behavioral cycles (McGowan et al., 2017) and enables the direct observation of habitat interactions and behaviors. We used radiotelemetry to test and refine our understanding of the beneficial interaction of shrubs with lizards. To our knowledge, incorporating radiotelemetry into a facilitation study is a novel use of the method.

We sought to test the hypothesis that shrubs facilitated lizards by providing thermoregulatory opportunity. We predicted that lizards would associate with shrubs for a meaningful proportion of their daily activity cycle; that shrub association would increase in the afternoon when daytime temperatures peak (Filazzola, Sotomayor, Sotomayor, & Lortie, 2017); and that lizard association with shrubs would be correlated with thermoregulatory behaviors. The results of our study confirm the application of radiotelemetry to ecological facilitation studies and the application of such studies to the description of beneficial interactions between shrubs and vertebrate ectotherms.

## 2 MATERIALS AND METHODS

### 2.1 Study site

**Figure 2:**
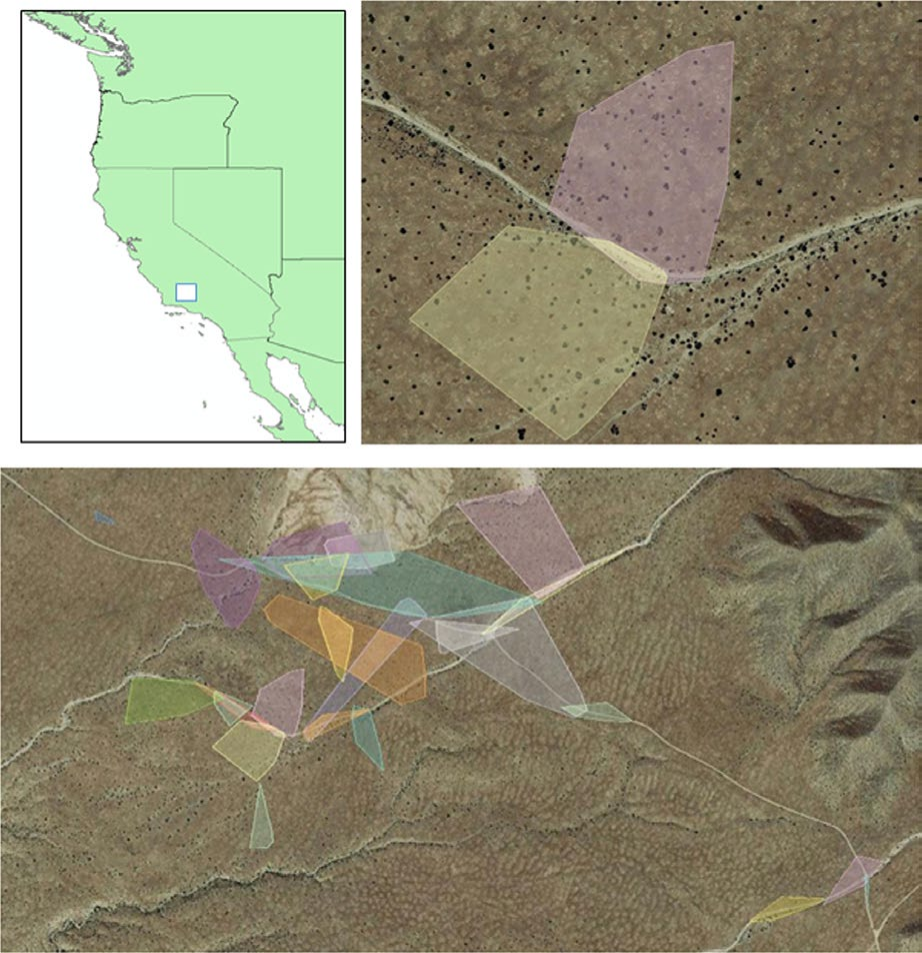
Study site on the Elkhorn Plain, Carrizo Plain National Monument, California. Top left: Location of study area within California. Top right: aerial photograph of study site overlain with sample home ranges calculated using a 95% minimum convex polygon (MCP) estimate, for each individual. Bottom: Aerial image depicting all home ranges of lizards in the study. Different individuals are indicated by different colors

The study was conducted on the Elkhorn Plain within Carrizo Plain National Monument (San Luis Obispo County, California, USA, 35.1914°N, 119.7929°W; Figure 2) within the San Joaquin Desert ecosystem (Germano et al., 2011). Average annual precipitation within the Monument ranges from 15 cm in the southeast to 25 cm in the northwest (Hijmans, Cameron, Parra, Jones, & Jarvis, 2005). The Elkhorn Plain is located within the Monument on an elevated plain separated from the main valley floor of the Carrizo Plain by the San Andreas Fault (Germano & Williams, 2005). The area has been heavily invaded by non‐native annual grasses including *Bromus madritensis, Erodium cicutarium*, and *Hordeum murinum* (Gurney, Prugh, & Brashares, 2015; Schiffman, 1994; Stout, Buck‐Diaz, Taylor, & Evens, 2014) but still provides habitat for endemic keystone species such as the giant kangaroo rat *Dipodomys ingens* (Bean et al., 2014). California jointfir, *Ephedra californica* was the dominant shrub at our study site. A much smaller woody perennial, *Gutierrezia californica*, can be found in some portions of the site at low frequency. The blunt‐nosed leopard lizard, *Gambelia sila*, was well documented on the study site (Germano, Smith, & Tabor, 2007).

### 2.2 Study species

*Ephedra californica*, a basal gymnosperm in the Gnetophyta division, is a large, slow‐growing woody shrub restricted to arid environments in western North America (Sawyer, Keeler‐Wolf, & Evens, 2009). Although the genus has a worldwide distribution and is represented by over a dozen species in the desert southwest of North America, *E. californica* is the only species that occurs in the San Joaquin Valley, where it is locally considered rare and sensitive (Sawyer et al., 2009) and has been documented to be a foundation species in the San Joaquin Desert community (Hawbecker, 1951; Lortie, Filazzola, & Westphal, 2017; Lortie, Liczner, et al., 2017). *Ephedra californica* is the only large shrub represented at our study site. *Gambelia sila* is a state and federally listed endangered species endemic to the San Joaquin Valley and restricted to San Joaquin Desert habitat (Germano & Rathbun, 2016; Germano & Williams, 1992; Germano et al., 2011; U.S. Fish & Wildlife Service, 1998; Warrick, Kato, & Rose, 1998). *Gambelia sila* are diurnal and mainly insectivorous though they may eat smaller lizard species on occasion (Germano et al., 2007; Warrick et al., 1998). Though *G. sila *can bury themselves and will occasionally dig primitive burrows, they mostly utilize abandoned burrows of other animals such as *D. ingens* (Fields, Coffin, & Gosz, 1999; Prugh & Brashares, 2012). Adult *G. sila *are inactive in burrows for much of the year, emerging only from late March or April through July (Germano & Rathbun, 2016; U.S. Fish & Wildlife Service, 1998; Warrick et al., 1998). During the active season, *G. sila* will also spend the night underground in burrows and may return to a burrow during the day if the temperature becomes too hot or cold (Germano & Rathbun, 2016; Warrick et al., 1998).

### 2.3 Experimental design

*Gambelia sila* individuals were located during foot and vehicle surveys and captured using a pole and noose. Individuals were collared following the method of Germano and Rathbun, (2016). VHF radio transmitters (Holohil model BD‐2, frequency 151–152 MHz, battery life 8–16 weeks, Holohil Systems Ltd., Carp, ON, Canada) were attached to a small beaded chain collar using jewelry wire and epoxy, and the collars were then fastened around the lizard's neck. *Gambelia sila* were kept overnight to ensure the collar was fitted correctly and did not irritate or harm the animal and were then released at their capture site. Collars weighed 1.6–2.2 g (depending on the size of chain needed for the lizard's neck), and we ensured that the weight of the collar did not exceed between 5% and 10% of the body mass of the individual.

In the first 2 days following release, all captured *G. sila* individuals were relocated (i.e., repeatedly sighted using radio telemetry) several times to ensure that the lizards were successfully adjusting to the collars and that impacts to their behavior and survival were minimal. We looked for any negative effects the collar had on the lizards, such as impacts on movement or any other deviation from normal lizard behaviors. *Gambelia sila *were then formally surveyed for 24 consecutive days. Surveys were conducted on each lizard 3 times a day. Two of these daily surveys were conducted during daylight hours, when lizards were typically active above ground. One survey was conducted before noon, and one was conducted after noon. The third survey was conducted during the night when lizards are inactive below ground. The “night survey” was conducted before 7:30 a.m. or after 7:30 p.m. on each day.

*Gambelia sila* were relocated using a 3‐element Yagi antenna and Model R‐100 telemetry receiver (Communications Specialists, Inc., Orange, CA, USA). Once found, a location was taken for each lizard using a Garmin 64st GPS unit (Garmin Ltd., Olathe, KS, USA) and a laser rangefinder (Bushnell Outdoor Products, Overland Park, KS, USA). Habitat was categorized as whether a lizard was within 0.5 m of a shrub (shrub) or not (open) (henceforth, the “shrub association zone”), and behavior was scored from a suite of predetermined behavioral syndromes (Supporting Information Table S1). Disturbance from the observer to the lizard was kept to a minimum for each observation to avoid influencing behavior and habitat selection. At the completion of the study, all collars were removed from the lizards.

### 2.4 Analyses

Analyses were conducted in R (version 3.3.2). Habitat association was analyzed using a generalized linear model (Bolker et al., 2009) with the multcomp package (Hothorn, Bretz, & Westfall, 2008). Behavioral data were analyzed with a multinomial logistic regression using the nnet package that accounts for the multiple levels of nominal outcomes of the observations (Venables & Ripley, 2002). Home range size was calculated using a 95% minimum convex polygon (MCP) estimation (Mohr, 1947) using the adehabitatHR package (Calenge, 2006). MCPs were visualized in two dimensions in R.

Shrub density was calculated by visually counting individual shrubs within each lizard's MCP using aerial photographs (Google Earth, image taken December 20, 2016, accessed November 2017) and dividing that number by the area in square meters of the MCP. We calculated a standardized measure of shrub association zone area using on‐the‐ground measurements of a randomly chosen sample of shrubs in the study area (*n* = 61), from which we calculated an average radius for each shrub following the method of Filazzola, Westphal, et al. (2017) and to which we added the 0.5 m association criterion described above. We calculated the area of each shrub association zone using the formula πr^2^ and then took the average across the sample. We multiplied this standardized shrub association zone area by the number of shrubs counted in each MCP to obtain an estimate of the percent area of an MCP subsumed by shrub association zones.

R code used for this project can be found at https://zenodo.org/record/1412857

## 3 RESULTS

A total of 28 lizards were relocated on five or more instances. On a given day, the median total number of relocations was 22 with a maximum of 27 and a minimum of 1 relocation for a total of 1,190 relocations.

On average the home ranges of the lizards overlapped with only two other individuals within a population throughout the entire sampling period (mode = 2 overlapping mcp polygons, one‐sample *t* test for µ = 2, *t* = −0.22535, *df* = 26, *p*‐value = 0.8235), and there were no significant differences between the two genders in the extent of overlapping number of home ranges (GLM, family = poisson with total area per individual as covariate, χ^2^ = 42.416, *p* = 0.39806). Our results were thus consistent with Tollestrup, (1983), Warrick et al., (1998) and Germano and Rathbun, (2016).

Mean female MCP area was 1.87 ha ± 0.53 *SE*. Mean male MCP area was 5.14 ha ± 2.15 *SE*. The difference in MCP area between males and females was not significant (Pr < Chi 0.095920). Gender was initially included as a factor in all other analyses but no relevant effects were significant (not reported); therefore, gender was subsequently removed from the remaining analyses.

### 3.1 Habitat

**Figure 3:**
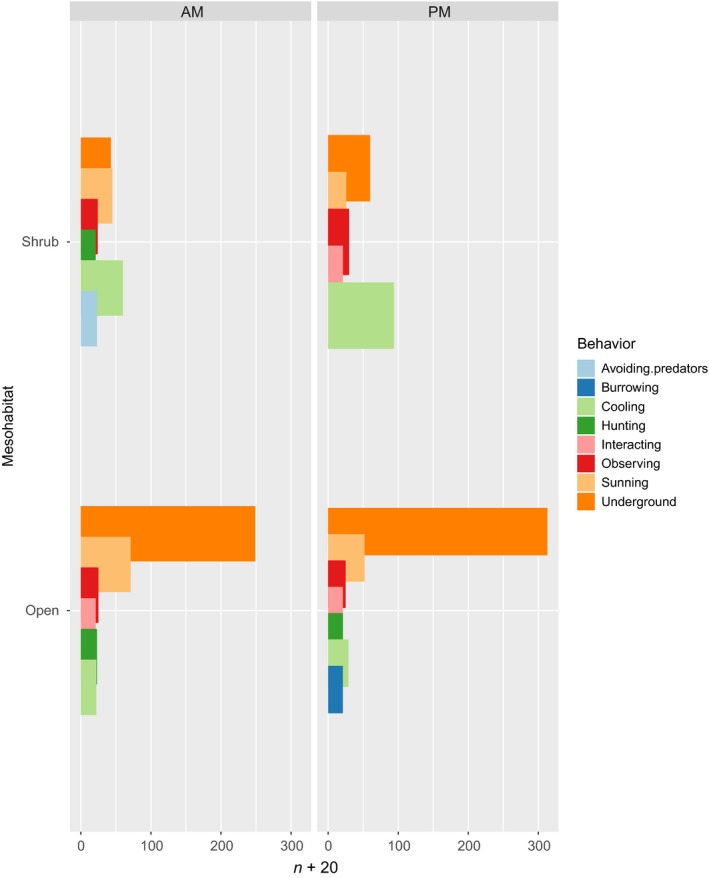
Plot of *Gambelia sila* behaviors with respect to habitat and time. Lizards engaged significantly more often in cooling behaviors when under shrubs during afternoon temperature peak. AM indicates observations were made between 0900 and 1300 hr; PM indicates observations were made between 1300 and 1700 hr

The frequency of lizard observations differed significantly between shrub and open (Figure 3, Table 1, *p* < 0.01). Shrub association frequencies of individual lizards ranged from 0 to 0.63 with a mean of 0.23 ± 0.035 *SE* (Supporting Information Table S2). Observations of lizards within open habitat did not differ between different times of day, but observations of lizards associating with shrubs differed significantly between morning and afternoon with lizards being found more frequently at shrubs in the afternoon (Table 1, *p* = 0.0252).

**Table 1:** Generalized linear model for habitat associated with relocated *Gambelia sila*, with degrees of freedom, deviance, and *p*‐values

Generalized linear model					
Factor	*df*	Deviance	*p*‐Value		
Habitat	1	88.33	<0.0001		
Time class	1	2.901	0.1		
Habitat:time.class	1	5.281	0.01		

Post hoc, least squared means					
Contrast	Estimate	*SE*	*df*	*z*.ratio	*p*.Value
Open, AM‐shrub, AM	0.769229	0.102934	NA	7.473	<0.0001
Open, AM‐open, PM	−0.01848	0.067966	NA	−0.272	0.993
Open, AM‐shrub, PM	0.44597	0.085189	NA	5.235	<0.0001
Shrub, AM‐open, PM	−0.78771	0.102727	NA	−7.668	<0.0001
Shrub, AM‐shrub, PM	−0.32326	0.11485	NA	−2.815	0.0252
Open, PM‐shrub, PM	0.464446	0.084938	NA	5.468	<0.0001

### 3.2 Behavior

Behavior differed significantly between habitat types (Figure 3, Table 2, *p* < 0.0001). Lizards were observed cooling under shrubs significantly more than in the open (Figure 3, Table 2, *p* < 0.0001). Because simple presence under shrubs may not necessarily imply cooling, we used cues from individual lizard postures when scoring their behavior as “cooling” (Supporting Information Table S1). Lizards were also observed avoiding predators under shrubs more frequently than at other microhabitat types (Table 2, *p* < 0.0001). The predators that lizards were observed avoiding in this study were all aerial predators (either ravens or raptor species). Predator interactions were all indirect and based on the observer's intuition; therefore, this result should be regarded as preliminary data. Burrowing and interacting occurred significantly less often under shrubs (*p* < 0.0001). Other types of behavior such as sunning, hunting, or active observation did not differ significantly between habitat types. Observed behavior also differed significantly between different times of day, for example, lizards were more frequently observed sunning in the morning in both habitat types compared to the afternoon and more often burrowing and avoiding predators in the afternoon (Figure 2, Table 2, *p* < 0.001).

**Table 2:** Multinomial logistic regression for observations of *Gambelia sila* behaviors associated with shrubs

Factor	Shrub		Time.class	
	*z*	*p*‐Value	*z*	*p*‐Value
Avoiding. predators	6.61E+01	<0.0001	4.60E+07	<0.0001
Burrowing	−1.88E+07	<0.0001	2.71E+01	<0.0001
Cooling	8.80E+00	<0.0001	1.65E+00	9.91E‐02
Hunting	8.27E‐01	0.4084232	−1.94E+00	5.23E‐02
Interacting	−1.74E+01	<0.0001	−8.19E‐01	4.13E‐01
Observing	1.14E+00	0.2534383	−8.04E‐01	4.21E‐01
Sunning	6.02E‐01	0.5468632	−6.51E+00	7.67E‐11

### 3.3 Shrub use as a function of shrub density and area

**Figure 4:**
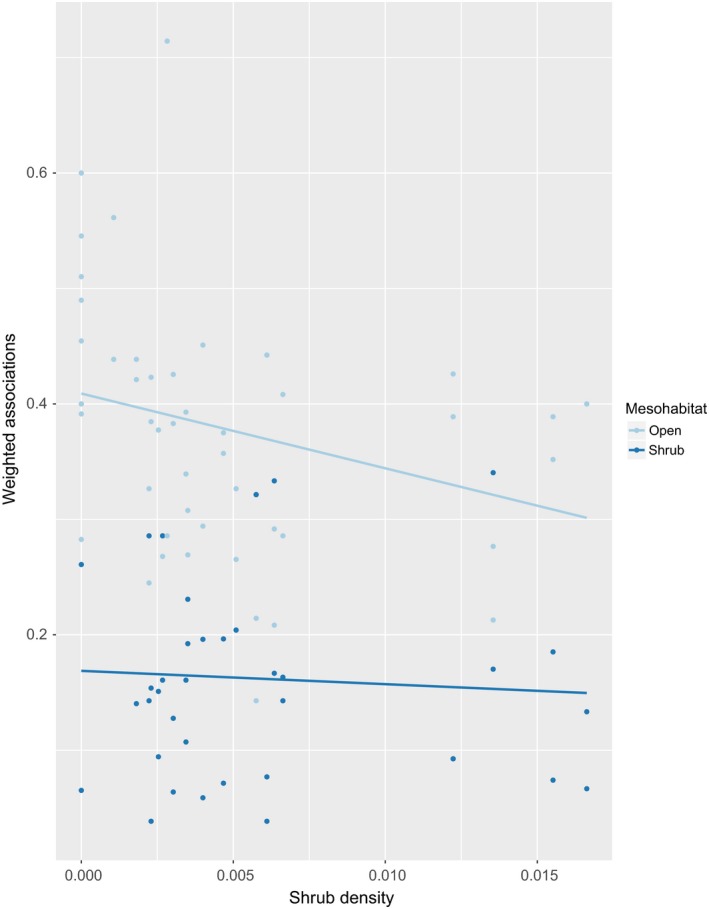
Plots of shrub density on the weighted *Gambelia sila* associations with shrubs

Shrub use by individual lizards did not vary significantly as a function of shrub density within that lizard's home range (Figure 4). Percent of MCP areas subsumed by shrub association zones ranged from 1% to 15% with an average of 5% of total surface area, and frequency of shrub use by lizards was significantly higher than predicted by the percent of MCP area subsumed by shrubs (*Z* = −4.714 from a Wilcoxon Signed ranks test, *p < *0.001).

## 4 DISCUSSION

Shrubs are foundation species in many ecosystems due to the facilitative benefits that they provide to both plant and animal species (Filazzola & Lortie, 2014; Lortie, Filazzola, & Sotomayor, 2016). We hypothesized that *E. californica* facilitates *G. sila* by providing thermoregulatory benefits. Our finding that individual lizards were associated with shrubs more than predicted by shrub area within their home ranges as well as the observed significant shift toward shrub association in the afternoon during peak daytime temperatures supports our hypothesis. Our behavioral observations provided a suite of potential behaviors that lizards were likely to perform during the observation period; thus, our finding that cooling predominated under shrubs compared to other behaviors further supports our hypothesis. Our observation that shrub use was not correlated with shrub density suggests that lizards are actively choosing shrubs over open habitats rather than as a consequence of shrubs being more densely distributed in their home ranges. The observed association of *G. sila* with shrubs is consistent with results of studies of thermoregulatory behavior of lizards (Sears et al., 2016, Vickers, Manicom, & Schwarzkopf, 2011, Vickers & Schwarzkopf 2016, Basson et al., 2017, Grimm‐Seyfarth, Mihoub, & Henl 2017) and suggests that shrubs may facilitate *G. sila* by providing shade. Although facilitation as an ecological process does not necessarily include lifetime fitness as a component (Stachowicz, 2001, Bruno et al., 2003, Michalet et al., 2011, Michalet & Pugnaire, 2016), our inference that *E. californica* facilitates *G. sila* would take on additional relevance, particularly to the potential for community structuring and the promotion of resilience in lizard populations, if the effects of *E. californica* facilitation on *G. sila* individual fitness were quantified. In the case of diurnal lizards, the link between thermal habitat quality and individual fitness has been firmly established by both theory and empirical testing (Kirchhof et al., 2018, Ortega, Mencía, & Pérez‐Mellado, 2016, Vickers et al., 2016, Pontes‐da‐Silva et al., 2018, Sinervo et al., 2018, Camacho et al., 2018), but we believe further study on the *E. californica*—*G. sila* relationship with respect to individual fitness is warranted.

Shrubs can buffer the extremes of multiple environmental conditions such as temperature, wind, and solar radiation, creating a moderate microclimate under their canopy (Kerr & Bull, 2004; Pugnaire, 2010). At the landscape scale, the presence of shrubs and their pattern of distribution (i.e., clumped vs. dispersed) will affect lizard thermoregulatory behavior and can be crucial to an ectotherm's thermoregulatory efficiency (Basson et al., 2017; Sears et al., 2016). Sources of shade are particularly important for ectotherms, which must maintain body temperature through behavior (Díaz & Cabezas‐Díaz, 2004; Huey, 1974; Huey & Slatkin, 1976; Kerr & Bull, 2004). Visual concealment from predators and physical protection is also important (Fields et al., 1999, Filazzola, Westphal, et al., 2017). Shrubs may therefore provide important mechanisms of facilitation for *G. sila*. Our results suggest an important mechanism (shrub restoration) for the management of desert ectotherms such as *G. sila* and provide support for radiotelemetry as a viable method for studying ecological facilitation.

Shrub use by *G. sila* was addressed in one previous paper that also used radiotelemetry. Germano and Rathbun, (2016) employed post hoc tests to answer the question of whether shrubs are important components of *G. sila* habitat. One test depended on an assumption based on Schoepf, Schmol, Keonig, Pillay, and Schradin (2015) that home ranges were resource‐based (i.e., shrub‐limited) and would thus be smaller in the presence of high‐quality habitat (=shrubs), while another test sought to bound the amount of shrub habitat present in lizard home ranges away from a null expectation. The authors found no effect of shrubs on home range size but did find more shrubs present within lizard home ranges than predicted. Our a priori approach (i.e., taking direct observations of association with shrubs) provided evidence that lizards actively seek out shrubs rather than randomly encountering them during their daily activity. The lack of a correlation between individual shrub use and shrub density suggests that a threshold presence of shrubs may be sufficient to provide thermoregulatory opportunity, therefore a strong correlation between absolute number of shrubs within a home range, and home range size would not be predicted. This conclusion is further supported by the Germano and Rathbun's (2016) result that home ranges tended to include more shrub habitat than predicted by the study‐site wide prediction, that is, it is likely beneficial that some shrubs be available within the daily activity theater of individual lizards. Our results therefore confirm and are consistent with the results from Germano and Rathbun, (2016).

Germano and Rathbun, (2016) also provide a caveat against overestimating the importance of shrubs to *G. sila* by noting that *G. sila* occurs in places that lack shrubs. Given the variation that we observed in lizard shrub association within one population is not surprising that entire populations can persist in relatively shrubless areas. It should be pointed out that the possibility that lizards in shrubless areas may be using alternative strategies to effectively thermoregulate (Germano & Rathbun, 2016 suggest rodent burrows may substitute for shrubs) the fact they may do so does not negate our findings that shrubs provide thermoregulatory benefits to lizards.

Although heritability of thermoregulatory response in lizards has been found to be low in species where it has been estimated (Logan et al., 2018; Paranjpe, Bastiaans, Patten, Cooper, & Sinervo, 2013), heritable variation in propensity to use shrubs could allow a population to adapt to the loss of shrubs at the landscape scale (presuming that shrubs were primordially present; Logan, Cox, & Calsbeek, 2014). However, where population‐scale variation exists in the predisposition to use shrubs, such as we found in this study, it would be reasonable to propose that shrubs be made available to those lizards that are predisposed to associate with shrubs. The net effect would be to optimize the habitat available for that population. Such optimization may be crucial to impart population resilience to climate change (Sears et al., 2016; Sinervo et al., 2010). Additionally, structured and/or heterogeneous habitats are becoming increasingly recognized as important to achieve individual‐scale thermoregulatory optimization for lizards (Basson et al., 2017; Clusella‐Trullas & Chown, 2014; Goller, Goller, & French, 2014; Sears et al., 2016).

## 5 CONCLUSIONS

Our results document the benefits of shrubs to vertebrate ectotherms in desert communities, including endangered species such as *G. sila*, thus providing guidance for land managers evaluating habitat preservation and restoration designs. We also advance methodology by demonstrating the utility of combining ecological facilitation theory with radiotelemetry. It should be noted that our study was not intended to test the hypothesis that *G. sila* require shrubs per se. Rather, we designed our study to ask whether shrubs provide benefits to *G. sila *and found evidence to support our hypothesis. In our view, this subtle divergence in focus and outcome demonstrates the power of taking an ecological facilitation approach to community interactions.

## CONFLICT OF INTEREST

None declared.

## AUTHOR'S CONTRIBUTIONS

MFW, CJL, and HSB acquired funding for the project; MFW, CJL, HSB, and TN conceived the study; TN and MFW collected the data; CJL, TN, and MFW analyzed the data; MFW, CJL, HSB and TN led the writing of the manuscript. All authors contributed critically to the drafting of the paper and gave final approval for publication.

## DATA ACCESSIBILITY

Data are available at https://zenodo.org/record/1412857 and at: *Code*: Lortie, C. J., T. Noble, S. Butterfield, and M. Westphal. R code and analyses testing desert shrub facilitation via radiotelemetric monitoring of a diurnal lizard. Zenodo, https://doi.org/10.5281/zenodo.1287938. https://zenodo.org/record/1287938#.WyAaFC2ZNhE. *Data*: Taylor Noble, Christopher Lortie, Scott Butterfield, and Michael Westphal. 2018. Radiotelemetric monitoring of a diurnal lizard in Carrizo National Monument. Knowledge Network for Biocomplexity. https://doi.org/10.5063/F1736P23.

## References

[ece34673-bib-0001] Adolph, S. C. (1990). Influence of behavioral thermoregulation on microhabitat use by two Sceloporus lizards. Ecology, 71, 315–327. 10.2307/1940271

[ece34673-bib-0002] Bachelet, D. , Ferschweiler, K. , Sheehan, T. , & Strittholt, J. (2016). Climate change effects on southern California deserts. Journal of Arid Environments, 127, 17–29. 10.1016/j.jaridenv.2015.10.003

[ece34673-bib-0003] Basson, C. H. , Levy, O. , Angilletta, M. J. , & Clusella‐Trullas, S. (2017). Lizards paid a greater opportunity cost to thermoregulate in a less heterogeneous environment. Functional Ecology, 31(4), 856–865. 10.1111/1365-2435.12795

[ece34673-bib-0004] Bean, W. T. , Prugh, L. R. , Stafford, R. , Butterfield, H. S. , Westphal, M. , & Brashares, J. S. (2014). Species distribution models of an endangered rodent offer conflicting measures of habitat quality at multiple scales. Journal of Applied Ecology, 51(4), 1116–1125. 10.1111/1365-2664.12281

[ece34673-bib-0005] Bolker, B. M. , Brooks, M. E. , Clark, C. J. , Geange, S. W. , Poulsen, J. R. , Stevens, M. H. H. , & White, J.‐S.‐S. (2009). Generalized linear mixed models: A practical guide for ecology and evolution. Trends in Ecology & Evolution, 24(3), 127–135. 10.1016/j.tree.2008.10.008 19185386

[ece34673-bib-0006] Bruno, J. F. , Stachowicz, J. J. , & Bertness, M. D. (2003). Inclusion of facilitation into ecological theory. Trends in Ecology and Evolution, 18, 119–125. 10.1016/S0169-5347(02)00045-9

[ece34673-bib-0007] Bulleri, F. , Bruno, J. F. , Silliman, B. R. , & Stachowicz, J. J. (2016). Facilitation and the niche: Implications for coexistence, range shifts and ecosystem functioning. Functional Ecology, 30(1), 70–78. 10.1111/1365-2435.12528

[ece34673-bib-0008] Calenge, C. (2006). The package adehabitat for the R software: A tool for the analysis of space and habitat use by animals. Ecological Modelling, 197, 516–519. 10.1016/j.ecolmodel.2006.03.017

[ece34673-bib-0009] Camacho, A. , Rusch, T. , Ray, G. , Telemeco, R. S. , Rodrigues, M. T. , & Angilletta, M. J. (2018). Measuring behavioral thermal tolerance to address hot topics in ecology, evolution, and conservation. Journal of Thermal Biology, 73, 71–79. 29549993 10.1016/j.jtherbio.2018.01.009

[ece34673-bib-0010] Clusella‐Trullas, S. , & Chown, S. L. (2014). Lizard thermal trait variation at multiple scales: A review. Journal of Comparative Physiology B: Biochemical, Systemic, and Environmental Physiology, 184, 5–21. 10.1007/s00360-013-0776-x 23989339

[ece34673-bib-0011] Cook, E. R. (2004). Long‐term aridity changes in the western United States. Science (New York, N.Y.), 306(5698), 1015–1018. 10.1126/science.1102586 15472040

[ece34673-bib-0012] Díaz, J. A. , & Cabezas‐Díaz, S. (2004). Seasonal variation in the contribution of different behavioural mechanisms to lizard thermoregulation. Functional Ecology, 18(6), 867–875. 10.1111/j.0269-8463.2004.00916.x

[ece34673-bib-0013] Fields, M. , Coffin, D. , & Gosz, J. (1999). Burrowing activities of kangaroo rats and patterns in plant species dominance at a shortgrass steppe‐desert grassland ecotone. Journal of Vegetation Science, 10(1), 123–130. 10.2307/3237167

[ece34673-bib-0014] Filazzola, A. , & Lortie, C. J. (2014). A systematic review and conceptual framework for the mechanistic pathways of nurse plants. Global Ecology and Biogeography, 23, 1335–1345. 10.1111/geb.12202

[ece34673-bib-0015] Filazzola, A. , Sotomayor, D. A. , & Lortie, C. J. (2017). Modelling the niche space of desert annuals needs to include positive interactions. Oikos, 127(2), 264–273. 10.1111/oik.04688

[ece34673-bib-0016] Filazzola, A. , Westphal, M. , Powers, M. , Liczner, A. R. , Smith Woollett, D. A. , Johnson, B. , & Lortie, C. J. (2017). Non‐trophic interactions in deserts: Facilitation, interference, and an endangered lizard species. Basic and Applied Ecology, 20, 51–61. 10.1016/j.baae.2017.01.002

[ece34673-bib-0017] Flores, J. , & Jurado, E. (2003). Are nurse‐protégé interactions more common among plants from arid environments? Journal of Vegetation Science, 14, 911–916. 10.1111/j.1654-1103.2003.tb02225.x

[ece34673-bib-0018] Germano, D. J. , & Rathbun, G. B. (2016). Home range and habitat use by blunt‐nosed leopard lizards in the southern San Joaquin Desert of California. Journal of Herpetology, 50(3), 429–434. 10.1670/15-006

[ece34673-bib-0019] Germano, D. J. , Rathbun, G. B. , Saslaw, L. R. , Cypher, B. L. , Cypher, E. A. , & Vredenburgh, L. M. (2011). The San Joaquin Desert of California: Ecologically misunderstood and overlooked. Natural Areas Journal, 31(2), 138–147. 10.3375/043.031.0206

[ece34673-bib-0020] Germano, D. J. , Smith, P. T. , & Tabor, S. P. (2007). Food habits of the blunt‐nosed leopard lizard (*Gambelia sila*). The Southwestern Naturalist, 52(2), 318–323. 10.1894/0038-4909(2007)52[318:FHOTBL]2.0.CO;2

[ece34673-bib-0021] Germano, D. J. , & Williams, D. F. (1992). Recovery of the blunt‐nosed leopard lizard: Past efforts, present knowledge, and future opportunities. Transactions of the Western Section of the Wildlife Society, 28, 38–47.

[ece34673-bib-0022] Germano, D. J. , & Williams, D. F. (2005). Population ecology of blunt‐nosed leopard lizards in high elevation foothill habitat. Journal of Herpetology, 39(1), 1–18. https://doi.org/10.1670/0022-1511(2005) 039[0001:PEOBLL]2.0.CO;2

[ece34673-bib-0023] Goller, M. , Goller, F. , & French, S. S. (2014). A heterogeneous thermal environment enables remarkable behavioral thermoregulation in *Uta stansburiana* . Ecology and Evolution, 4, 3319–3329. 25535549 10.1002/ece3.1141 PMC4228607

[ece34673-bib-0024] Grimm-Seyfarth, A. , Mihoub, J.-B. , & Henl, K. (2017). Too hot to die? The effects of vegetation shading on past, present, and future activity budgets of two diurnal skinks from arid Australia. Ecology & Evolution, 7, 6803–6813. 10.1002/ece3.3238 28904761 PMC5587462

[ece34673-bib-0025] Gurney, C. M. , Prugh, L. R. , & Brashares, J. S. (2015). Restoration of native plants is reduced by rodent‐caused soil disturbance and seed removal. Rangeland Ecology and Management, 68(4), 359–366. 10.1016/j.rama.2015.05.001

[ece34673-bib-0026] Hannah, L. , Carr, J. L. , & Lankerani, A. (1995). Human disturbance and natural habitat: A biome level analysis of a global data set. Biodiversity and Conservation, 4, 128–155. 10.1007/BF00137781

[ece34673-bib-0027] Hawbecker, A. C. (1951). Small mammal relationships in an *Ephedra* community. Journal of Mammalogy, 32(1), 50–60.

[ece34673-bib-0028] Hijmans, R. J. , Cameron, S. E. , Parra, J. L. , Jones, P. G. , & Jarvis, A. (2005). Very high resolution interpolated climate surfaces for global land areas. International Journal of Climatology, 25(15), 1965–1978. 10.1002/joc.1276

[ece34673-bib-0029] Hoekstra, J. M. , Boucher, T. M. , Ricketts, T. H. , & Roberts, C. (2005). Confronting a biome crisis: Global disparities of habitat loss and protection. Ecology Letters, 8(1), 23–29. 10.1111/j.1461-0248.2004.00686.x

[ece34673-bib-0030] Hothorn, T. , Bretz, F. , & Westfall, P. (2008). Simultaneous inference in general parametric models. Biometrical Journal, 50, 346–363. 10.1002/bimj.200810425 18481363

[ece34673-bib-0031] Huey, R. B. (1974). Behavioral thermoregulation in lizards: Importance of associated costs. Science, 184(4140), 1001–1003. 10.1126/science.184.4140.1001 4826166

[ece34673-bib-0032] Huey, R. B. , & Slatkin, M. (1976). Cost and benefits of lizard thermoregulation. The Quarterly Review of Biology, 51(3), 363–384. 10.1086/409470 981504

[ece34673-bib-0033] Kearney, M. , Shine, R. , & Porter, W. P. (2009). The potential for behavioral thermoregulation to buffer “cold‐blooded” animals against climate warming. Proceedings of the National Academy of Sciences of the United States of America, 106(10), 3835–3840. 10.1073/pnas.0808913106 19234117 PMC2656166

[ece34673-bib-0034] Kéfi, S. , Rietkerk, M. , Alados, C. L. , Pueyo, Y. , Papanastasis, V. P. , ElAich, A. , & de Ruiter, P. C. (2007). Spatial vegetation patterns and imminent desertification in Mediterranean arid ecosystems. Nature, 449(7159), 213–217. 10.1038/nature06111 17851524

[ece34673-bib-0035] Kerr, G. D. , & Bull, C. M. (2004). Microhabitat use by the scincid lizard *Tiliqua rugosa*: Exploiting natural temperature gradients beneath plant canopies. Journal of Herpetology, 38(4), 536–545. 10.1670/82-04A

[ece34673-bib-0036] Kirchhof, S. , Hetem, R. S. , Lease, H. M. , Miles, D. , Mitchell, D. , Müller, J. , … Murray, I. W. (2018). Thermoregulatory behaviour and high thermal preference buffer impact of climate change in a Namib Desert lizard. Ecosphere, 8(12), e02033 10.1002/ecs2.2033

[ece34673-bib-0037] Logan, M. L. , Cox, R. M. , & Calsbeek, R. (2014). Natural selection on thermal performance in a novel thermal environment. Proceedings of the National Academy of Sciences of the United States of America, 111(39), 14165–14169. 10.1073/pnas.1404885111 25225361 PMC4191752

[ece34673-bib-0038] Logan, M. L. , Curlis, J. D. , Gilbert, A. L. , Miles, D. B. , Chung, A. K. , McGlothlin, J. W. , & Cox, R. M. (2018). Thermal physiology and thermoregulatory behaviour exhibit low heritability despite genetic divergence between lizard populations. Proceedings of the Royal Society B, 285, 20180697 10.1098/rspb.2018.0697 29743257 PMC5966615

[ece34673-bib-0039] Lortie, C. J. , Filazzola, A. , & Sotomayor, D. A. (2016). Functional assessment of animal interactions with shrub‐facilitation complexes: A formal synthesis and conceptual framework. Functional Ecology, 30(1), 41–51. 10.1111/1365-2435.12530

[ece34673-bib-0040] Lortie, C. , Filazzola, A. , & Westphal, M. (2017). The foundation species effect of *Ephedra californica* . Knowledge Network for Biocomplexity. 10.5063/F1VM49D1

[ece34673-bib-0041] Lortie, C. J. , Liczner, A. , Filazzola, A. , Noble, T. , Gruber, E. , & Westphal, M. F. (2017). The Groot Effect: Plant facilitation and desert shrub regrowth following extensive damage. Ecology and Evolution, 2017, 1–10. 10.1002/ece3.3671 PMC5756850 29321907

[ece34673-bib-0042] McGowan, J. , Beger, M. , Lewison, R. L. , Harcourt, R. , Campbell, H. , Priest, M. , … Possingham, H. P. (2017). Integrating research using animal‐borne telemetry with the needs of conservation management. Journal of Applied Ecology, 54(2), 423–429. 10.1111/1365-2664.12755

[ece34673-bib-0043] Mcintire, E. J. B. , & Fajardo, A. (2014). Facilitation as a ubiquitous driver of biodiversity. New Phytologist, 201(2), 403–416. 10.1111/nph.12478 24102266

[ece34673-bib-0044] Michalet, R. , & Pugnaire, F. I. (2016). Facilitation in communities: Underlying mechanisms, community and ecosystem implications. Functional Ecology, 30, 3–9. 10.1111/1365-2435.12602

[ece34673-bib-0045] Michalet, R. , Xiao, S. , Touzard, B. , Smith, D. S. , Cavieres, L. A. , Callaway, R. M. , & Whitham, T. G. (2011). Phenotypic variation in nurse traits and community feedbacks define an alpine community. Ecology Letters, 14, 433–443. 10.1111/j.1461-0248.2011.01605.x 21366815

[ece34673-bib-0046] Mohr, C. O. (1947). Table of equivalent populations of North American small mammals. American Midland Naturalist, 37(1), 223 10.2307/2421652

[ece34673-bib-0047] Mouat, D. A. , & Lancaster, J. M. (2008). *Drylands in crisis* . Environmental Change and Human Security (pp. 67–80).

[ece34673-bib-0048] Ortega, Z. , Mencía, A. , & Pérez‐Mellado, V. (2016). Behavioral buffering of global warming in a cold‐adapted lizard. Ecology & Evolution, 6, 4582–4590. 10.1002/ece3.2216 27386098 PMC4931003

[ece34673-bib-0049] Paranjpe, D. A. , Bastiaans, E. , Patten, A. , Cooper, R. D. , & Sinervo, B. (2013). Evidence of maternal effects on temperature preference in side‐blotched lizards: Implications for evolutionary response to climate change. Ecology and Evolution, 3(7), 1977–1991. 10.1002/ece3.614 23919144 PMC3728939

[ece34673-bib-0050] Pontes‐da‐Silva, E. , Magnusson, W. E. , Sinervo, B. , Caetano, G. H. , Miles, D. B. , Colli, G. R. , … Werneck, F. P. (2018). Extinction risks forced by climatic change and intraspecific variation in the thermal physiology of a tropical lizard. Journal of Thermal Biology, 73, 50–60. 10.1016/j.jtherbio.2018.01.013 29549991

[ece34673-bib-0051] Prugh, L. R. , & Brashares, J. S. (2012). Partitioning the effects of an ecosystem engineer: Kangaroo rats control community structure via multiple pathways. Journal of Animal Ecology, 81(3), 667–678. 10.1111/j.1365-2656.2011.01930.x 22098534

[ece34673-bib-0052] Pugnaire F. I. (Ed.) (2010). Positive plant interactions and community dynamics. Boca Raton, FL: CRC Press.

[ece34673-bib-0053] Sawyer, J. , Keeler‐Wolf, T. , & Evens, J. (2009). A manual of California vegetation (2nd ed., p. 1300). Sacramento, CA: California Native Plant Society.

[ece34673-bib-0054] Schiffman, P. M. (1994). Promotion of exotic weed establishment by endangered giant kangaroo rats (*Dipodomys ingens*) in a California grassland. Biodiversity and Conservation, 3(6), 524–537. 10.1007/BF00115158

[ece34673-bib-0055] Schoepf, I. , Schmol, G. , Keonig, B. , Pillay, N. , & Schradin, C. (2015). Manipulation of population density and food availability affects home range sizes of African striped mouse females. Animal Behaviour, 99, 53–60. 10.1016/j.anbehav.2014.10.002

[ece34673-bib-0056] Sears, M. W. , & Angilletta, M. J. (2015). Costs and benefits of thermoregulation revisited: Both the heterogeneity and spatial structure of temperature drive energetic costs. The American Naturalist, 185(4), E94–E102. 10.1086/680008 25811092

[ece34673-bib-0057] Sears, M. W. , Angilletta, M. J. , Schuler, M. S. , Borchert, J. , Dilliplane, K. F. , Stegman, M. , … Mitchell, W. A. (2016). Configuration of the thermal landscape determines thermoregulatory performance of ectotherms. Proceedings of the National Academy of Sciences of the United States of America, 113(38), 10595–10600. 10.1073/pnas.1604824113 27601639 PMC5035910

[ece34673-bib-0058] Sinervo, B. , Mendez‐de‐la‐Cruz, F. , Miles, D. B. , Heulin, B. , Bastiaans, E. , Villagran‐Santa Cruz, M. , … Sites, J. W. (2010). Erosion of lizard diversity by climate change and altered thermal niches. Science, 328(5980), 894–899. 10.1126/science.1184695 20466932

[ece34673-bib-0059] Sinervo, B. , Miles, D. B. , Wu, Y. , Méndez de la Cruz, F. R. , Kirchoff, S. , & Qi, Y. (2018). Climate change, thermal niches, extinction risk and maternal‐effect rescue of toad‐headed lizards, *Phrynocephalus*, in thermal extremes of the Arabian Peninsula to the Tibetan Plateau. Integrative Zoology, 13, 450–470. 10.1111/1749-4877.12315 29436768

[ece34673-bib-0060] Stachowicz, J. J. (2001). Mutualisms, positive interactions, and the structure of ecological communities. BioScience, 51, 235–246.

[ece34673-bib-0061] Stout, D. , Buck‐Diaz, J. , Taylor, S. , & Evens, J. (2014). Vegetation mapping and accuracy assessment report for Carrizo Plain National Monument. California Native Plants Society. Retrieved from https://www.cnps.org/cnps/vegetation/pdf/carrizo-mapping_rpt2013.pdf. Accessed January 21, 2017.

[ece34673-bib-0062] Tollestrup, K. (1983). Growth and reproduction in two closely related species of leopard lizards, Gambelia silus and Gambelia wislizenii. American Midland Naturalist, 108, 1–20.

[ece34673-bib-0063] U.S. Fish and Wildlife Service (1998). Recovery Plan for Upland Species of the San Joaquin Valley, California (pp. 1–319). Portland, OR.

[ece34673-bib-0064] Venables, W. N. , & Ripley, B. D. (2002). Modern applied statistics with S (4th ed.). New York, NY: Springer.

[ece34673-bib-0065] Vickers, M. , Manicom, C. , & Schwarzkopf, L. (2011). Extending the cost‐benefit model of thermoregulation: High‐temperature environments. The American Naturalist, 177(4), 452–461. 10.1086/658150 21460567

[ece34673-bib-0066] Vickers, M. , & Schwarzkopf, L. (2016). A simple method to predict body temperature of small reptiles from environmental temperature. Ecology and Evolution, 6, 3059–3066. 27252829 10.1002/ece3.1961 PMC4870193

[ece34673-bib-0067] Warrick, G. D. , Kato, T. T. , & Rose, B. R. (1998). Microhabitat use and home range characteristics of blunt‐nosed leopard lizards. Journal of Herpetology, 32(2), 183–191. 10.2307/1565295

[ece34673-bib-0068] Westphal, M. F. , Stewart, J. A. E. , Tennant, E. N. , Butterfield, H. S. , & Sinervo, B. (2016). Contemporary drought and future effects of climate change on the endangered blunt‐nosed leopard lizard, *Gambelia sila* . PLoS ONE, 11(5), e0154838 10.1371/journal.pone.0154838 27136458 PMC4852947

