# Supplementary material for: A test of desert shrub facilitation via radiotelemetric monitoring of a diurnal lizard
Source: Ecol Evol. 2018 Nov 16;8(23):12153–62. doi: 10.1002/ece3.4673 (PMC6303751; doi:10.1002/ece3.4673)
Supplement: Supplementary file 1 [file ECE3-8-12153-s001.docx]

**Table S1**: Behavior classification table for lizard observations.

| Classification | Observed behavior |
| --- | --- |
| avoiding predators | Moving (most often running) away from predators |
| burrowing | Actively digging a burrow, or burying itself. This classification was only used if the lizards was actively creating its own burrow, it was not used if a pre-existing burrow was utilized. |
| cooling | Lizard moving into, or remaining still in shade and exhibiting following behaviors: sitting upright in shade with front legs extended and rear toes pointed up and off the ground; tail off the groundl. |
| hunting | Actively stalking or attempting to catch prey. Usually comprised of a slow stalking of an insect and then a sudden burst of speed. |
| interacting | Interacting with another lizard including either genders of the same species as well as members of other lizard species such as whiptail lizards (*Aspidocelis tigrinum*). |
| observing | Actively observing environment (eg moving head or body to track motion). |
| underground | Lizard underground, behavior could not be otherwise be determined. |
| sunning | Lizard in sun, not moving. Most often either low to ground, with lower body touching ground or sitting upright with head and shoulders up and rear toes pointed out. Eyes often closed or squinted. |
