# Supplementary material for: A test of desert shrub facilitation via radiotelemetric monitoring of a diurnal lizard
Source: Ecol Evol. 2018 Nov 16;8(23):12153–62. doi: 10.1002/ece3.4673 (PMC6303751; doi:10.1002/ece3.4673)
Supplement: Supplementary file 2 [file ECE3-8-12153-s002.docx]

**Table S2**. Frequencies of shrub association of individual lizard, density of shrubs within individual home ranges, and percent of area of individual Minimum Convex Polygons (MCP) subsumed by shrub association zones for radiotracked *Gambelia sila* on Elkhorn Plain in 2016.

| **Lizard ID** | **Total obs.** | **Shrub obs.** | **Shrub association**  **frequency** | | **MCP area m^2^** | **Number of shrubs** | **Shrub density** | **Shrub assoc area m^2^** | **% Shrub assoc area within MCP** |
| --- | --- | --- | --- | --- | --- | --- | --- | --- | --- |
| **180** | 59 | 0 | 0.000 | 7509 | | 8 | 0.001 | 73.491 | 0.010 |
| **320** | 5 | 0 | 0.000 | 2 | | 0 | 0.000 | 0.000 | 0.000 |
| **360** | 5 | 0 | 0.000 | 8 | | 0 | 0.000 | 0.000 | 0.000 |
| **500** | 50 | 0 | 0.000 | 803 | | 0 | 0.000 | 0.000 | 0.000 |
| **900** | 9 | 0 | 0.000 | 354 | | 1 | 0.003 | 9.186 | 0.026 |
| **939** | 12 | 0 | 0.000 | 9 | | 0 | 0.000 | 0.000 | 0.000 |
| **780** | 55 | 5 | 0.091 | 8190 | | 50 | 0.006 | 459.318 | 0.056 |
| **220** | 56 | 7 | 0.125 | 57400 | | 104 | 0.002 | 955.381 | 0.017 |
| **740** | 31 | 4 | 0.129 | 5636 | | 7 | 0.001 | 64.304 | 0.011 |
| **919** | 47 | 8 | 0.170 | 40998 | | 124 | 0.003 | 1139.108 | 0.028 |
| **381** | 17 | 3 | 0.176 | 5533 | | 92 | 0.017 | 845.144 | 0.153 |
| **439** | 55 | 10 | 0.182 | 3597 | | 44 | 0.012 | 404.200 | 0.112 |
| **760** | 52 | 10 | 0.192 | 4356 | | 10 | 0.002 | 91.864 | 0.021 |
| **660** | 50 | 11 | 0.220 | 5500 | | 22 | 0.004 | 202.100 | 0.037 |
| **960** | 54 | 12 | 0.222 | 18920 | | 48 | 0.003 | 440.945 | 0.023 |
| **520** | 53 | 12 | 0.226 | 4120 | | 5 | 0.001 | 45.932 | 0.011 |
| **820** | 55 | 14 | 0.255 | 3802 | | 59 | 0.016 | 541.995 | 0.143 |
| **840** | 55 | 14 | 0.255 | 10693 | | 50 | 0.005 | 459.318 | 0.043 |
| **240** | 57 | 15 | 0.263 | 21786 | | 75 | 0.003 | 688.976 | 0.032 |
| **979** | 50 | 16 | 0.320 | 13723 | | 91 | 0.007 | 835.958 | 0.061 |
| **0** | 46 | 15 | 0.326 | 20331 | | 88 | 0.004 | 808.399 | 0.040 |
| **800** | 28 | 11 | 0.393 | 7136 | | 25 | 0.004 | 229.659 | 0.032 |
| **540** | 50 | 20 | 0.400 | 15908 | | 81 | 0.005 | 744.095 | 0.047 |
| **420** | 50 | 21 | 0.420 | 4488 | | 10 | 0.002 | 91.864 | 0.020 |
| **680** | 56 | 24 | 0.429 | 51618 | | 138 | 0.003 | 1267.717 | 0.025 |
| **860** | 51 | 25 | 0.490 | 12759 | | 81 | 0.006 | 744.095 | 0.058 |
| **460** | 50 | 26 | 0.520 | 3838 | | 52 | 0.014 | 477.690 | 0.124 |
| **717** | 56 | 36 | 0.643 | 39292 | | 226 | 0.006 | 2076.116 | 0.053 |
